# Inhibitory effects of berberine on proinflammatory M1 macrophage polarization through interfering with the interaction between TLR4 and MyD88

**DOI:** 10.1186/s12906-019-2710-6

**Published:** 2019-11-19

**Authors:** Jing Gong, Jingbin Li, Hui Dong, Guang Chen, Xin Qin, Meilin Hu, Fen Yuan, Ke Fang, Dingkun Wang, Shujun Jiang, Yan Zhao, Wenya Huang, Zhaoyi Huang, Fuer Lu

**Affiliations:** 1 Department of Integrated Traditional Chinese and Western Medicine, Tongji Hospital, Tongji Medical College, Huazhong University of Science and Technology, Wuhan, 430030 Hubei China; 2 Institute of Integrated Traditional Chinese and Western Medicine, Tongji Hospital, Tongji Medical College, Huazhong University of Science and Technology, Wuhan, 430030 Hubei China

**Keywords:** Berberine, Macrophage, Inflammatory polarization, Lipopolysaccharide

## Abstract

**Backgrounds:**

Inflammation is recognized as the key pathological mechanism of type 2 diabetes. The hypoglyceamic effects of berberine (BBR) are related to the inhibition of the inflammatory response, but the mechanism is not completely clear.

**Methods:**

The inflammatory polarization of Raw264.7 cells and primary peritoneal macrophages were induced by LPS, and then effects and underlying mechanisms of BBR were explored. An inflammatory model was established by LPS treatment at different concentrations for different treatment time. An ELISA assay was used to detect the secretions of TNF-α. RT-PCR was applied to detect M1 inflammatory factors. The F4/80^+^ ratio and CD11c^+^ ratio of primary peritoneal macrophages were determined by flow cytometry. The expressions of p-AMPK and TLR4 were detected by Western blot. The cytoplasmic and nuclear distributions of NFκB p65 were observed by confocal microscopy. The binding of TLR4 to MyD88 was tested by CoIP, and the affinity of BBR for TLR4 was assessed by molecular docking.

**Results:**

Upon exposure to LPS, the secretion of TNF-α and transcription of inflammatory factors in macrophages increased, cell morphology changed and protrusions appeared gradually, the proportion of F4/80^+^CD11c^+^ M1 macrophages increased, and the nuclear distribution of NFκB p65 increased. BBR pretreatment partially inhibited the changes mentioned above. However, the expression of TLR4 and p-AMPK did not change significantly after LPS intervention for 3 h. Meanwhile, CoIP showed that the interaction between TLR4 and MyD88 increased, and BBR inhibited the binding. Molecular docking suggested that BBR might interact with TLR4.

**Conclusions:**

Inflammatory changes were induced in macrophages after LPS stimulation for 3 h, and BBR pretreatment inhibited inflammatory polarization. BBR might interact with TLR4 and disturb TLR4/MyD88/NFκB signalling pathway, and it might be the mechanism by which BBR attenuated inflammation in the early phase.

## Background

Inflammation is the key pathological mechanism of type 2 diabetes (T2DM) [1]. The infiltration of immune cells and the activation of inflammatory signals in adipose tissue have been found to be important characteristics of T2DM [2]. The inflammatory cells in the adipose tissue of obese mice produced cytokines, including IL-6, TNF-α, IL-1β and macrophage migration inhibitory factor [3]. More than 100 years ago, researchers observed that salicylate, the main metabolite of the anti-inflammatory drug aspirin, was beneficial for the blood glucose levels of patients with T2DM [4]. In 1993, Hotamisligil and colleagues found that TNF-α secreted by inflammatory cells in the adipose tissue of obese mice inhibited insulin signal transduction [5]. Subsequent clinical trials showed that inflammatory markers in the circulation indicated the risk of T2DM, plasma TNF-α and IL-6 concentrations in patients with T2DM increased, and the anti-inflammatory medicine salicylate could improve the metabolic imbalance [6–8]. In obese mice, the neutralization of TNF-α or IL-1β could reduce inflammation and improve insulin signalling [9].

Macrophages are the main sources of the inflammatory mediators TNF-α and IL-6 in the pathogenesis of T2DM [10, 11]. In obese and T2DM patients and animal models, monocytes in circulation are recruited to the adipose tissue, liver, pancreas, skeletal muscle and brain [12], and the accumulation of macrophages lead to local inflammation, islet β cell dysfunction and insulin resistance [13–15]. Under different metabolic conditions, macrophage populations differ in number, and phenotypic and functional changes also appear in metabolic tissues. According to surface markers and cytokine secretion, macrophages can be roughly divided into M1 and M2 macrophages [16–18]. For example, in the adipose tissue of healthy people, the macrophages are mainly the M2 type, while in obese and T2DM patients, M1 macrophges increase. M1 macrophages mainly secrete inflammatory cytokines, such as TNF-α, IL-1β and IL-6, which induce insulin resistance; M2 macrophages express anti-inflammatory factors, increase the differentiation of islet β cells and improve insulin signalling [19]. Inhibiting M1 polarization has become a promising strategy for the prevention and treatment of T2DM.

Berberine (BBR) was found to reduce the proportion of macrophages in the intestinal immune system [20] and inhibit the expression of inflammatory cytokines such as TNF-α, IL-1β and MIP1 in T2DM rats, and the inhibition of inflammation by BBR was related to the TLR4/MyD88/NFκB signalling pathway [21]. This study further explored the mechanism by which BBR inhibited the M1 macrophage polarization.

## Materials and methods

### Cell source and culture medium

Raw264.7 cells were gifts from the Union Hospital of Tongji Medical College, Huazhong University of Science and Technology. Kunming female mice weighing between 30 and 40 g were purchased from Hubei Disease Control Center and were used for the extraction of primary peritoneal macrophages. The animal ethics committee of Huazhong University of Science and Technology approved the study.

### Culture medium

Complete medium containing 10 ml of Gibco fetal bovine serum (FBS), 1 ml penicillin (100 units/ml) and streptomycin (100 mg/ml) and 89 ml of high-glucose DMEM was prepared. This complete medium was employed for cell culture and passage.

Synchronous medium, containing 1 ml of Gibco FBS, 1 ml of penicillin and streptomycin and 98 ml high-glucose DMEM, was used for reagent configuration, cell synchronization and intervention in Raw264.7 cells.

### Raw264.7 cell culture conditions and treatment

Cells were incubated at 37 °C with 5% CO2/95% air in complete medium. When the cells reached 50–60% confluence rate, they were subcultured. Before drug intervention, the cells were pre-incubated in synchronous medium for 12 h; then exposed to corresponding agents for the scheduled time.

### Extraction method of primary peritoneal macrophages

Female Kunming mice were sacrificed by cervical spine dislocation, soaked in 75% alcohol for 10 min and fixed. Then, the outer peritoneum was gentle sectioned and sufficiently separated from the inner layer. After the inner layer of the peritoneum was exposed, 12 ml of physiological saline was slowly injected into the abdominal cavity under the centre of the abdomen. After gentle shaking for 5 min, the peritoneal rinse liquid was slowly extracted from both sides of the peritoneum [22]. The collected cells were placed on ice. The extractions were repeated three times for each mouse. After centrifugation, cells were inoculated into 6-well or 12-well plates, and the medium was changed 30 min later. Following incubation for 6 h, the agent intervention was initiated.

### Preparation of LPS

A total of 10 mg of LPS was dissolved in 10 ml of an aseptic PBS solution and gently shaken until completely dissolution. The LPS solution was vibrated for 30 min and was stored at − 20 °C. LPS was diluted to different concentrations, namely, 10^− 4^, 10^− 5^, 10^− 6^, 10^− 7^ and 10^− 8^ mg/ml. Each dilution was vortexed for 10 min before being used.

### Preparation of BBR, metformin, AICAR and compound C

BBR was dissolved in the methanol-ethanol solution at the concentration of 3.345 mg/mL. After sterilization by filtration, and the stock solution of 0.01 M BBR was sealed, protected from light and stored at 4 °C. The BBR stock solution was diluted with synchronized culture medium to obtain various working solutions. A total of 33.12 mg metformin was dissolved in 2 ml of synchronous medium, filtered for sterility, and stored at − 20 °C. Synchronous medium was used to dilute the stock solution of metformin to the appropriate concentration for use in the experiment. 5-Aminoimidazole-4-carboxamide1-β-D-ribofuranoside (AICAR) and compound C were prepared according to the manuscripturer’s instructions. Pretreatment with BBR, metformin and other agents were applied before LPS intervention.

### MTT assay

Raw264.7 cells in the logarithmic phase were collected, and the cell suspension concentration was adjusted. One hundred microliter medium containing 6000 cells was added to each well of a 96-well plate. After incubation for 12 h, BBR, metformin, AICAR or Compound C were added according to the corresponding concentration gradient. In addition, the normal control group and blank well were set up. The supernatant was discarded 24 h later, and the wells were washed once with PBS. MTT solution dissolved in high-glucose DMEM was administered for 4 h. The supernatant of each well was carefully removed, and 100 μl of DMSO was then added to the wells for 30 min. A spectrophotometer (Bio-Tek, USA, Model: Synergy2) was used to measure the absorbance at 490 nm, and the cell survival rate was calculated.

### ELISA assay

A TNF-α ELISA kit (Boster, China) was used to detect the TNF-α concentrations in the supernatant. In brief, 100 μl of supernatant was added to each well according to the instructions, and the corresponding OD values were measured at 450 nm using a spectrophotometer (Synergy2, USA). The TNF-α levels were adjusted according to the cell number in each well.

### RT-PCR

RNA was extracted from the cells by Trizol reagent, the RNA concentration was determined, and reverse transcriptase kits were used to obtain cDNA. RNA concentration was examined by Nanodrop spectrophotometer (Thermo Scientific, America), and the samples with A260/A280 in the range of 1.8 to 2.0 were used for further study. Subsquently, a SYBR premix EX TaqTM kit (Takara, China) was used for amplification on a StepOne PCR detetor (Stepone, USA). The mRNA expression levels were calculated using 2^-Δ ΔCT^ method. The primer sequences are shown in Table 1.

**Table 1:** Primers in RT-PCR

Gene	Forward (5′ → 3′)	Reverse (5′ → 3′)
LBP	CCCAGACGCTGGATGTGATG	TGATCTGAGATGGCAAAGTAGACC
CD14	CTTATGCTCGGCTTGTTGCTGT	TAGCAGCGGACACTTTCCTCGT
IL-1β	GTCGGGACATAGTTGACTTCAC	GACTTGGCAGAGGACTTCAC
TNF-α	CCAGGTTCTCTTCAAGGGACAA	GGTATGAAATGGCAAATCGGCT
IL-10	GCTGGACAACATACTGCTGAC	AATGCTCCTTGATTTCTGGG
IL-4	CTGTCACCCTGTTCTGCTTTCTC	TTTCTGTGACCTGGTTCAAAGTGT
TGF-β	AAGGACCTGGGTTGGAAGT	CGGGTTGTGTTGGTTGTAGA
IL-6	AATCTGCTCTGGTCTTCTGGA	CAGTATTGCTCTGAATGACTCTGG
MIP1	GACTTTTAGTGGCACGAGCG	GCTTGCTGTAGTTGCGGTTCT
TNFR	ACCCTCACACTCACAAACCA	ATAGCAAATCGGCTGACGGT
β-actin	AGCCATGTACGTAGCCATCC	CTCTCAGCTGTGGTGGTGAA

### Western blot analysis

Proteins were extracted from the cells and separated by gel electrophoresis, transferred onto PVDF membranes, and incubated with antibodies against TLR4 (Abcam, UK), p-AMPK (CST, USA), and AMPK (CST, USA). Then, these membranes were co-incubated with corresponding secondary antibodies. The protein levels were determined by an Odyssey imaging system (LI-COR Biosciences, Lincoln, NE).

### Immunofluorescence assay

Cells were seeded on glass coverslips in a 12-well plate at a density of 5 × 10^5^ cells per well, and paraformaldehyde fixation was performed after treatment. Afterwards, these cells were immunostained overnight with primary antibodies against NFkB p65 (CST, USA). Then fluorescent secondary antibodies were co-incubated at room temperature for 30 min. After being rinsed, the cells were stained with DAPI (Sigma, USA) for 3 min and sealed. Finally, the cells were observed and imaged under a laser confocal microscope (Japan, Nikon, C2+/C2si+).

### CoIP

Approximately 10^7^ cells were seeded in each 6-cm dish and exposed to LPS and/or BBR for 3 h. Then, protein was extracted as described above and immunoprecipitated with anti-MyD88 for 1 h at 4 °C. The samples were incubated with protein A/G-Sepharose overnight at 4 °C under constant rotation and then washed with CoIP wash buffer 4 times [23]. Western blotting was used as described above to detect the levels of TLR4 and MyD88 in the immunoprecipitated samples.

### Determination of the ratio of peritoneal macrophages by flow cytometry

Cells were collected after treatment and then incubated with F4/80 (eBioscience, USA) or CD11c (BD Bioscience, USA) antibody in the dark for 30 min. The reaction was terminated with PBS, and the cells were fixed using 1% paraformaldehyde after rinsing. The M1 ratio was tested by flow cytometry (BD FACSCalibur, USA).

### Molecular docking

The PubChem and UniPROtkb databases were searched to obtain the structure of BBR and the PDB ID of TLR4. The protein structure of TLR4 was obtained from the RCSB PDB database. Chimera (http://www.cgl.ucsf.edu/chimera/) and Avogardora (https://avogadro.cc/) software were used to optimize the structure of BBR. After optimizing the TLR4 structure by Autodock software (http://www.scripps.edu/mb/olson/doc/autodock), the 3D active centres in the A chain, B chain, C chain and D chain of TLR4 were docked with BBR separately. During the simulation, the ligands were allowed to rotate freely; based on the probe atoms of the corresponding ligand atoms, the AutoGrid module was used to detect the active region of the target protein and generate a potential energy map. Through docking analysis, whether BBR can interact with the active centre of TLR4 was assessed.

### Statistical analysis

SPSS 20.0 software was used to analyse the data. The K-S normality test was performed to evaluate the homogeneity of variance, and ANOVA was used to determine significance. Significance between different groups was assessed using the Bonferroni test. Differences were considered significant when the *P* value < 0.05.

## Results

### Effects of intervention agents on the survival rate of Raw264.7 cells

**Fig. 1:**
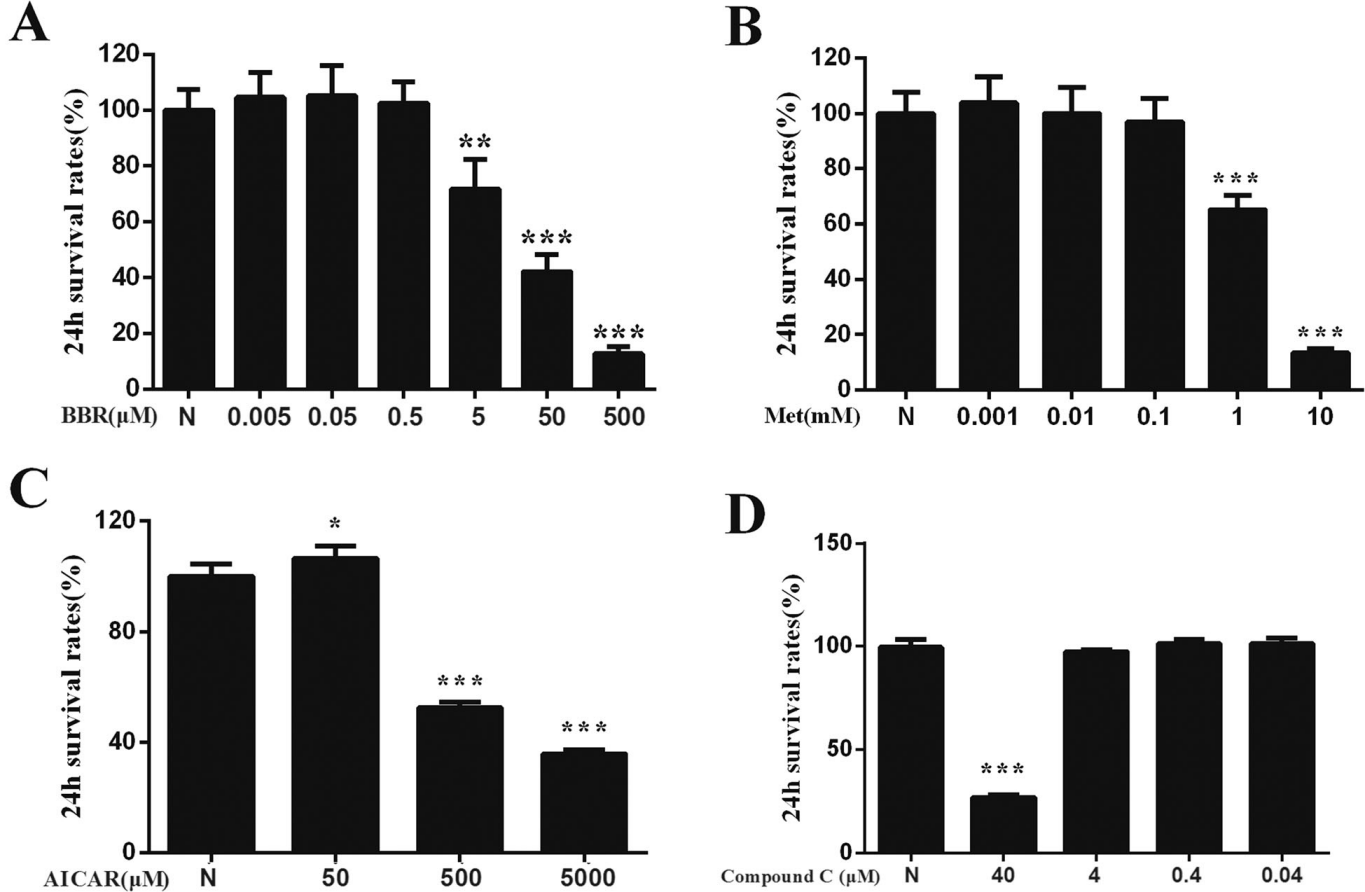
Effects of intervention agents on the survival rate of Raw264.7 cells. **a** BBR, berberine. **b** Met, metformin. **c** AICAR. **d** Compound C. N, control group. Compared with N group, *P < 0.05, **P < 0.01, ***P < 0.001

BBR, metformin, AICAR, and compound C were prepared in synchronous culture medium. Different concentrations of these agents were added to Raw264.7 cells for 24 h, and the cell survival rates were compared. Combined with previous research results, the maximum concentration that did not induce significant cytotoxicity was set as the concentration for subsequent experiments. As shown in Fig. 1, the intervention concentration of BBR was 0.5 μM, the concentration of metformin was 0.1 mM, the concentration of AICAR was 50 μM, and the concentration of Compound C was 4 μM.

### Identification of primary peritoneal macrophages

**Fig. 2:**
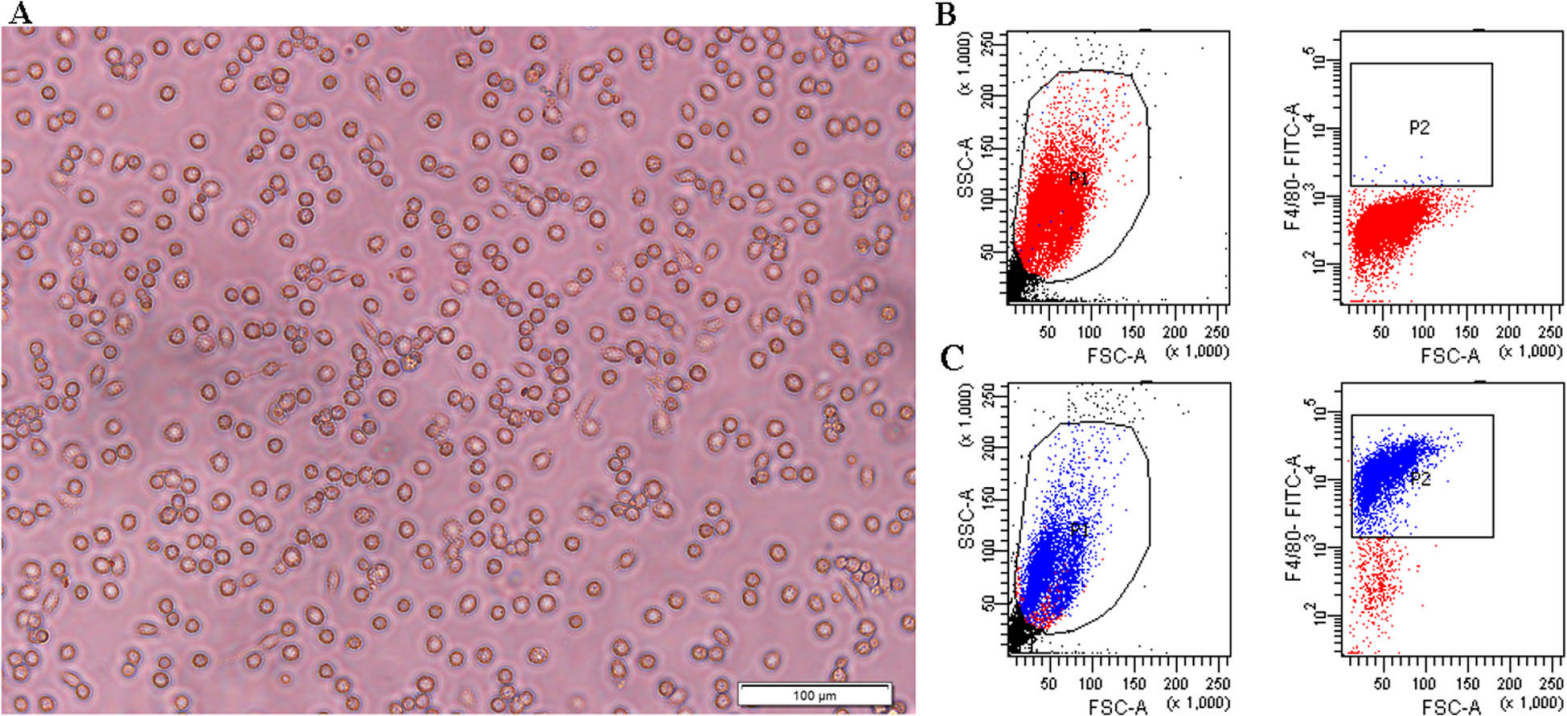
Peritoneal macrophage morphology (**a**) and proportion identification (**b**-**c**). **b** Negative controls without antibodies. **c** Macrophages labelled by F4/80

Peritoneal macrophage morphology and ratios were evaluated. Under light microscopy, the peritoneal macrophages extracted from the mice were round and scattered with a few fusiform fibroblasts (Fig. 2a). Macrophage ratio was determined by FITC-labelled F4/80. As shown in Fig. 2b-c, the macrophage ratio in the peritoneal lavage fluid was about 90%.

### Effects of LPS on morphology and TNF-α secretion in Raw264.7 macrophages

**Fig. 3:**
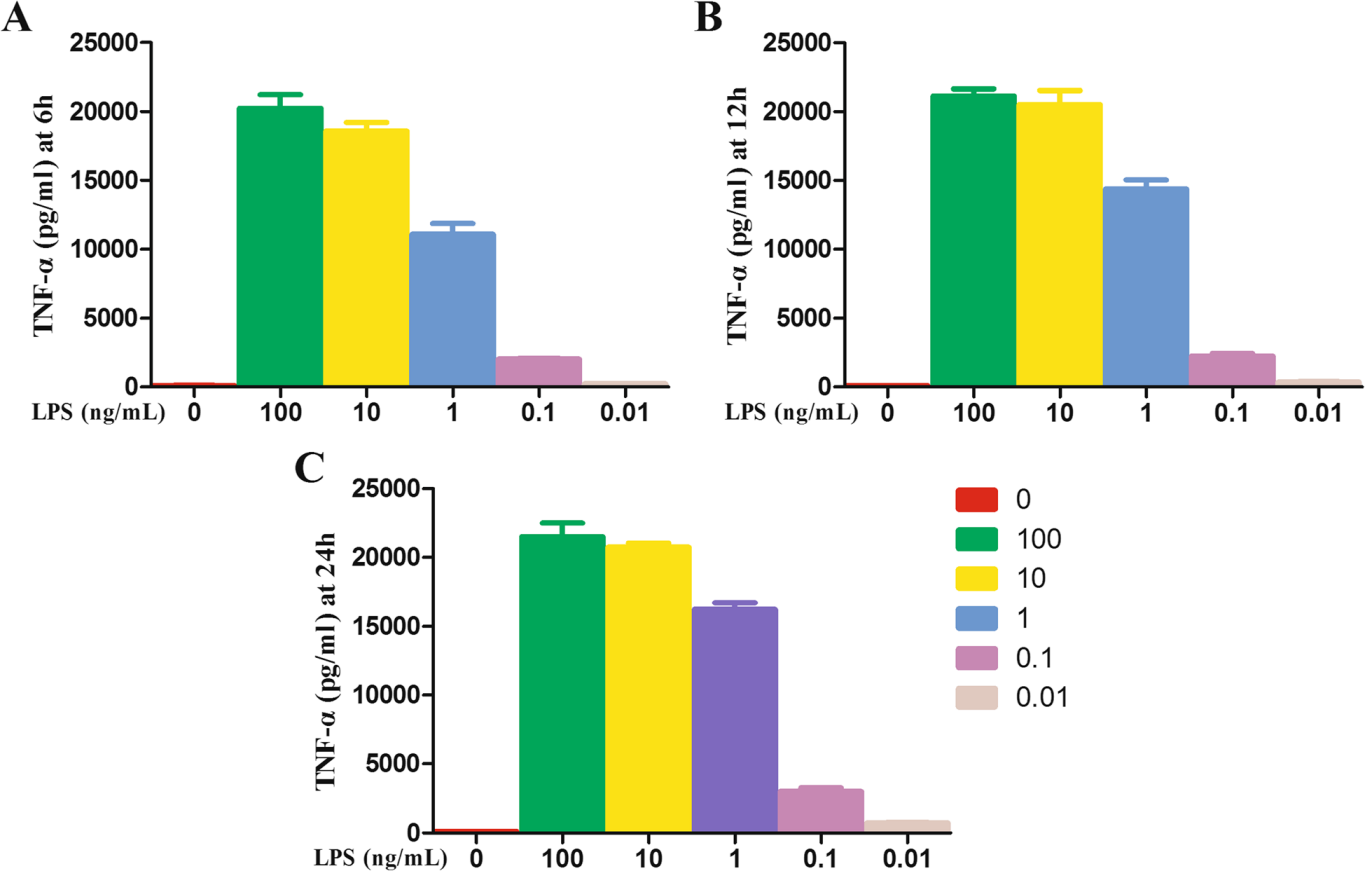
TNF-α levels in the supernatant after Raw264.7 cells were exposed to LPS at different concentrations for 6h (**a**), 12 h (**b**) or 24 h (**c**)

When Raw264.7 cells were exposed to 100, 10, 1, 0.1 or 0.01 ng/ml LPS for 6 h, 12 h or 24 h, the inflammatory TNF-α in the cell supernatant were significantly increased (Fig. 3). Concentration of 100 ng/ml and 0.1 ng/ml were then chosen as the high and low treatment doses for the subsequent experiment (LH, high dose of LPS; LL, low dose of LPS).

**Fig. 4:**
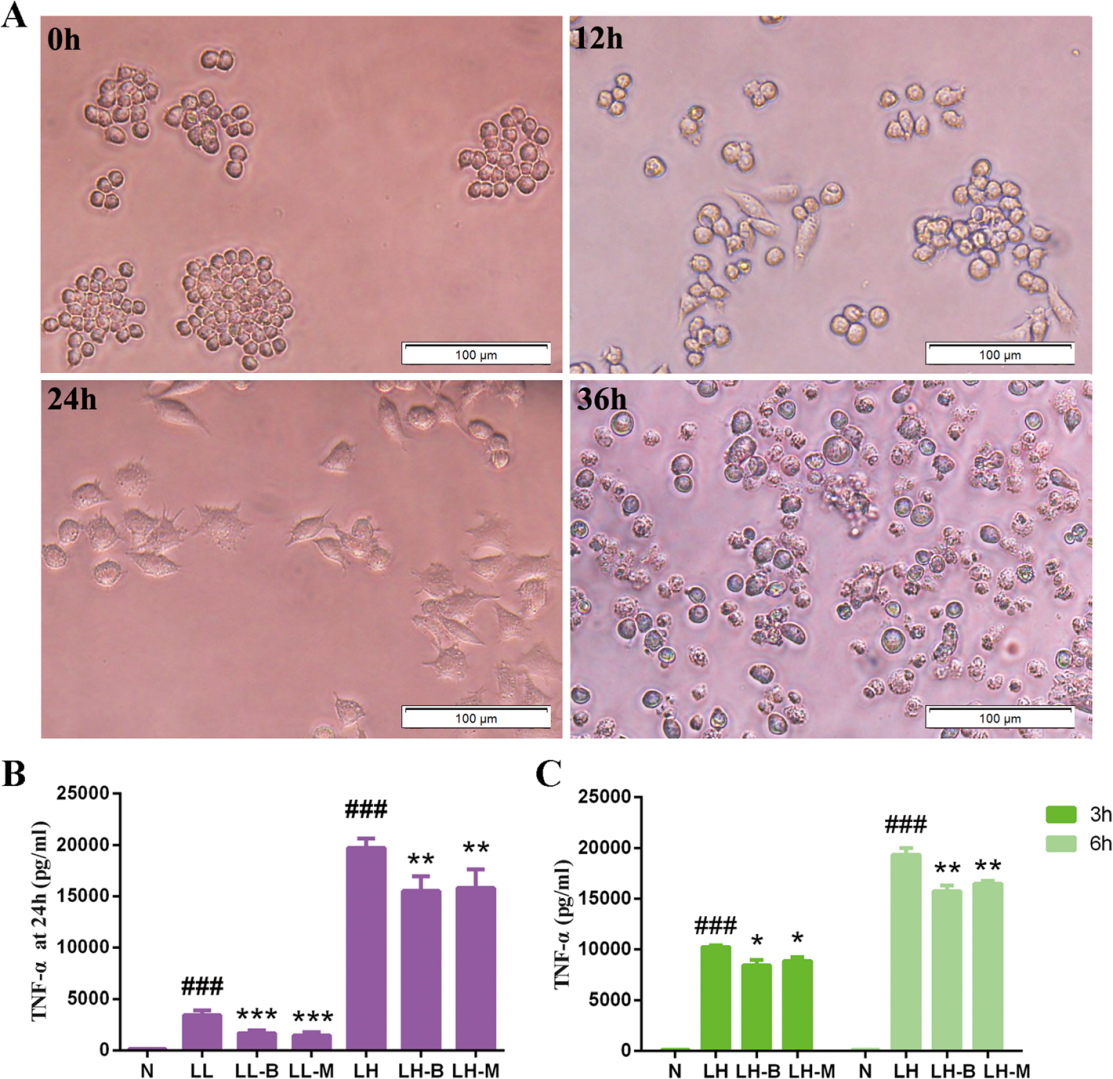
Morphological changes after high-dose LPS intervention (**a**) and the effects of BBR on LPS-induced TNF-α secretion (**b**-**c**) in Raw264.7 cells at different times. N, control group; LL, 0.1 μM LPS group; LL-B, 0.1 μM LPS + BBR group; LL-M, 0.1 μM LPS + Metformin group; LH, 0.1 mM LPS group; LH-B, 0.1 mM LPS + BBR group; LH-M, 0.1 μM LPS + Metformin group. Compared with control group, ^###^*P* < 0.001; Compared with LL or LH group, *P < 0.05, **P < 0.01, ***P < 0.001

In addition to the changes in TNF-α secretion, the Raw264.7 cells gradually became larger when exposed to high or low doses of LPS. With the prolongation of the intervention time, the cells especially those in the high dose group, extended several protrusions. After exposure to a high dose of LPS for 36 h, a large number of fragmentations were observed, and death occurred in the cells (Fig. 4a).

### Effects of BBR on inflammation induced by LPS in macrophages

BBR inhibited the secretion of TNF-α in Raw264.7 cells after intervention with LPS for 3 h and 24 h (Fig. 4b-c), and BBR did not significantly improve the morphological changes in Raw264.7 cells induced by high dose LPS.

**Fig. 5:**
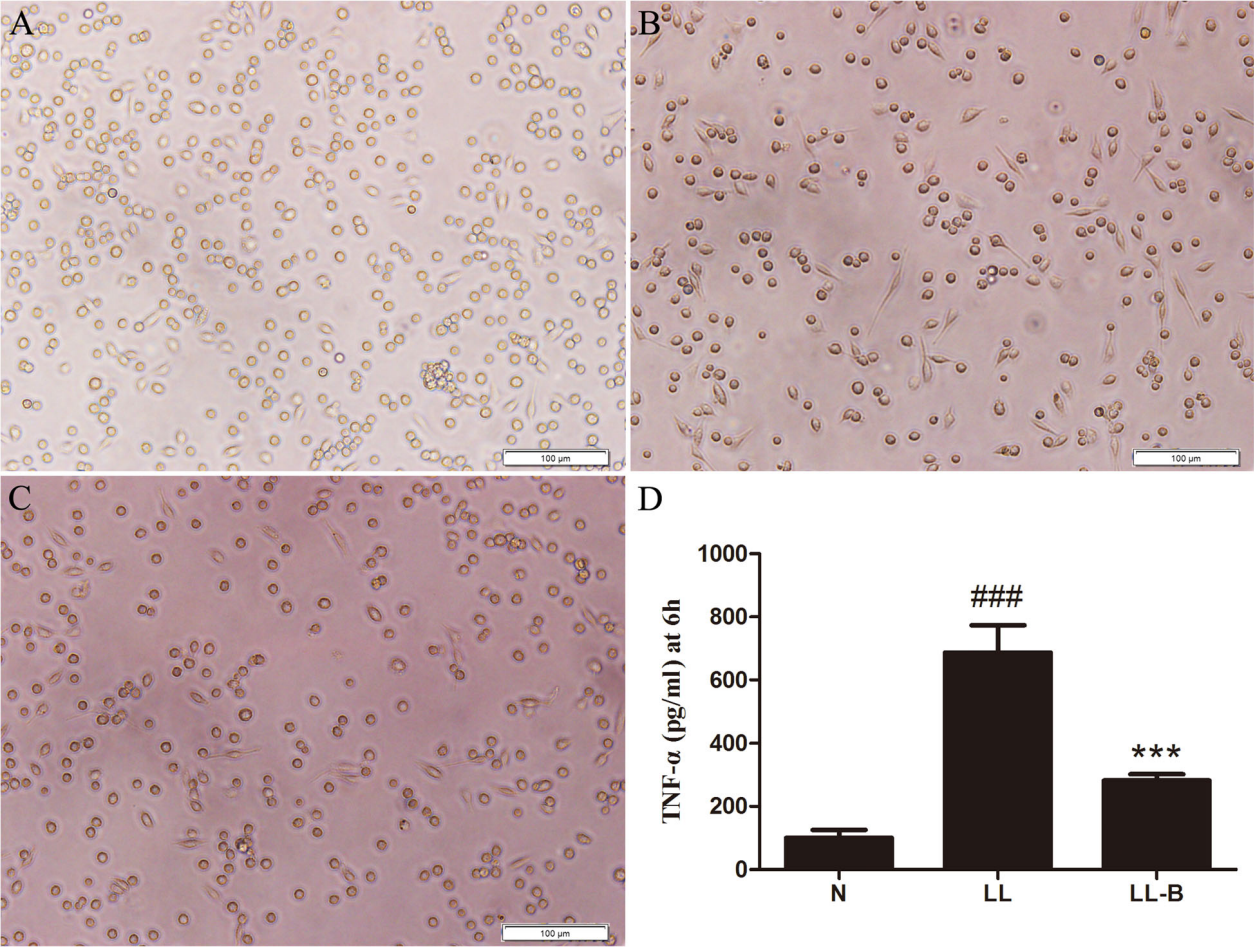
Effects of BBR on LPS-induced inflammation in peritoneal macrophages. The LPS intervention time was 6 h. **a** Control group. **b** 0.1 μg/ml LPS group. **c** 0.1 μg/ml LPS + BBR group. **d** Level of TNF-α in the supernatant. N, normal control group. LL, Low LPS group; LL-B, Low LPS + BBR group; Compared with N group, ^###^*P* < 0.001; Compared with LL group, ***P < 0.001

After treatment with 0.1 μg/ml LPS for 6 h, some peritoneal macrophages extended their protrusions, and the secretion of TNF-α in the supernatant increased. However, BBR pretreatment inhibited the morphological changes induced by LPS and the secretion of TNF-α in the supernatant (Fig. 5).

### Effects of BBR on M1 polarization induced by LPS

**Fig. 6:**
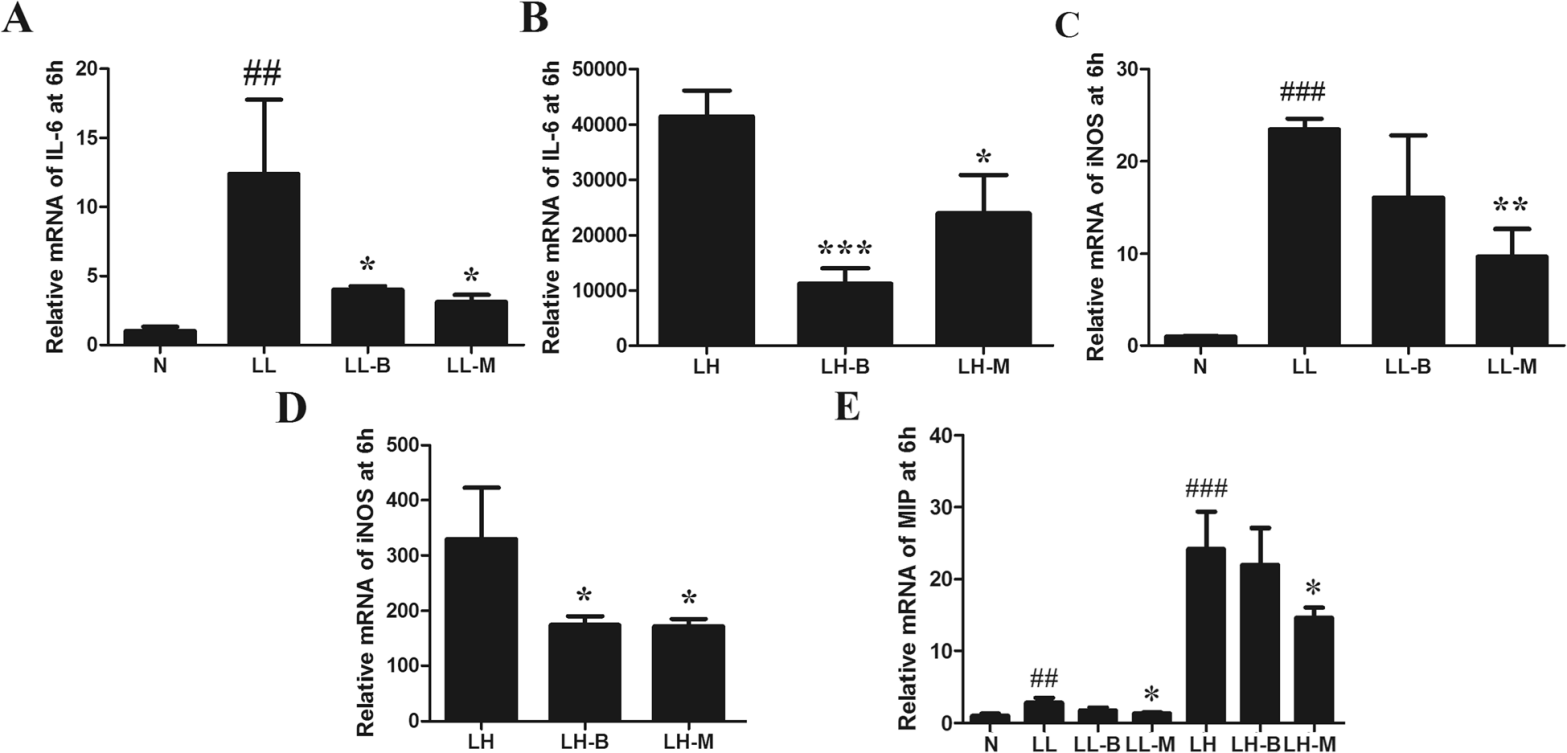
Effects of BBR on LPS-induced IL-6 (**a**-**b**), iNOS (**c**-**d**) and MIP1 (**e**) expressions in Raw264.7 cells. N, normal control group; LL, low LPS group; LL-B, low LPS + BBR group; LL-M, low LPS + Metformin group; LH, high LPS group; LH-B, high LPS + BBR group; LH-M, high LPS + Metformin group. Compared with N group, ^##^*P* < 0.01, ^###^*P* < 0.001; Compared with LL or LH group, *P < 0.05, **P < 0.01, ***P < 0.001

**Fig. 7:**
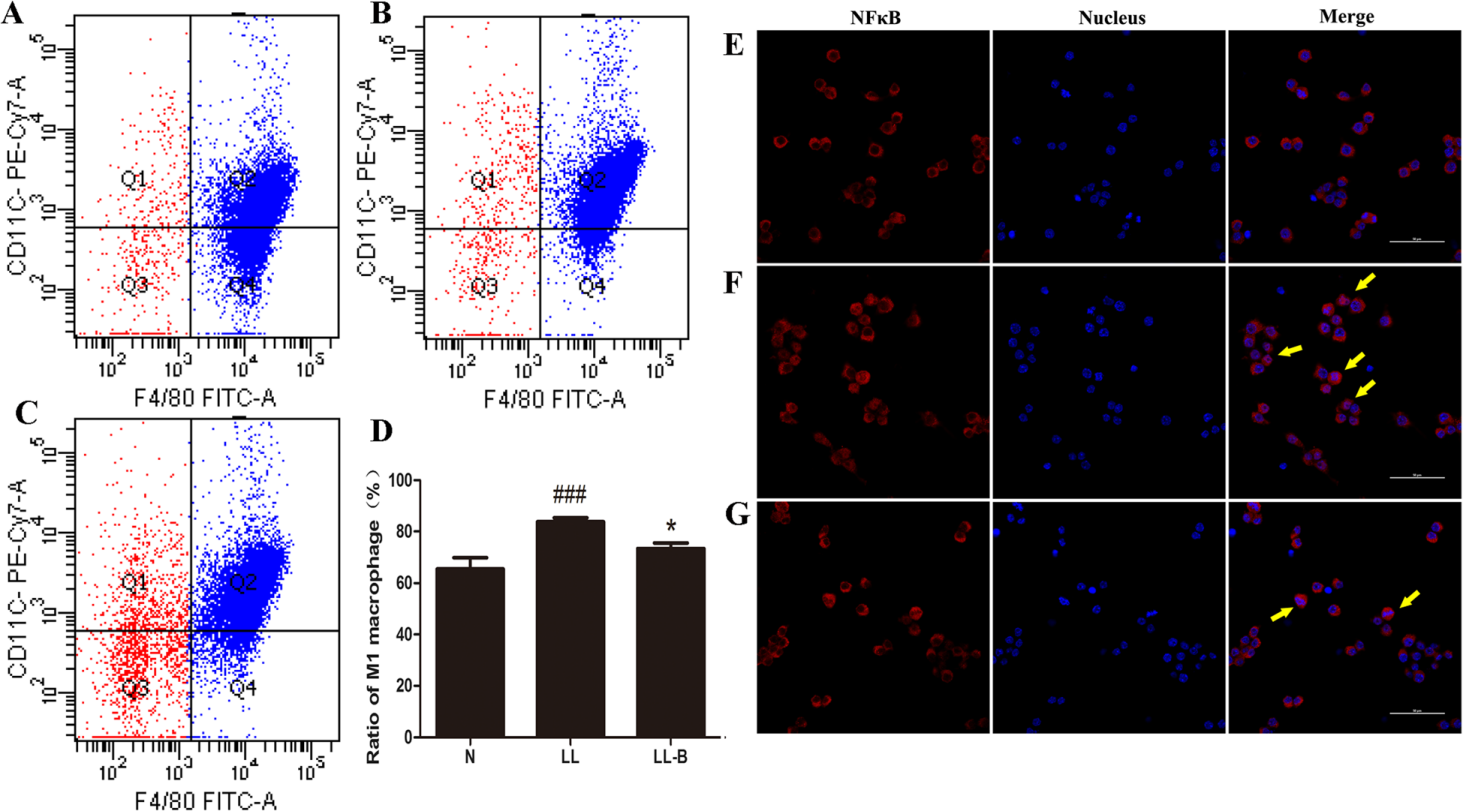
Effects of BBR on the M1 ratio (**a**-**d**) and nuclear translocation of NFκB (**e**-**g**) after LPS intervention for 3 h in primary peritoneal macrophages. **a** CD11c^+^ ratio in normal control group. **b** CD11c^+^ ratio in Low LPS group. **c** CD11c^+^ ratio in low LPS + BBR group. **d** Compared with N group, ^###^*P* < 0.001; compared with LL group, *P < 0.05. **e** Nucear transloacation of NFκB p65 in normal control group. **f** Nucear transloacation of NFκB p65 in LL group. **g** Nucear transloacation of NFκB p65 in LPS + BBR group. Red, NFκB p65; blue, nucleus; purplish red, NFκB p65 in nucleus. Yellow arrows, marked nucear transloacation of p65 NFκB

After LPS treatment, the transcription of IL-6, TNF-α and MIP1 increased, and iNOS2 expression was upregulated. BBR pretreatment inhibited the upregulation of LPS-induced inflammatory markers in Raw264.7 macrophages (Fig. 6). After LPS intervention, the proportion of CD11c^+^ M1 polarized macrophages was upregulated, while BBR pretreatment inhibited the increase in F4/80^+^CD11c^+^ peritoneal cells, decreasing the numbers of inflammatory polarized macrophages (Fig. 7a-d).

### Effects of BBR on the expressions of regulatory proteins related to macrophage polarization

The M1 polarization of macrophages was regulated by signalling molecules, such as the components of the TLR4/MyD88/NFκB pathway, TNFR, AMPK α1 and other TLRs, among which the components of the TLR4 signalling pathway were the most important. Thus, the protein and mRNA expression levels of these inflammatory signal molecules were detected. As mentioned above, after LPS intervention for 3 h and 6 h, TNF-α secretion was significantly increased, and meanwhile BBR inhibited the expression of inflammatory factors. Immunofluorescence also showed that the nuclear transloacation of NfκB p65 was significantly increased, and BBR decreased the level of NFκB p65 in the nucleus after LPS intervention for 3 h (Fig. 7e-g).

**Fig. 8:**
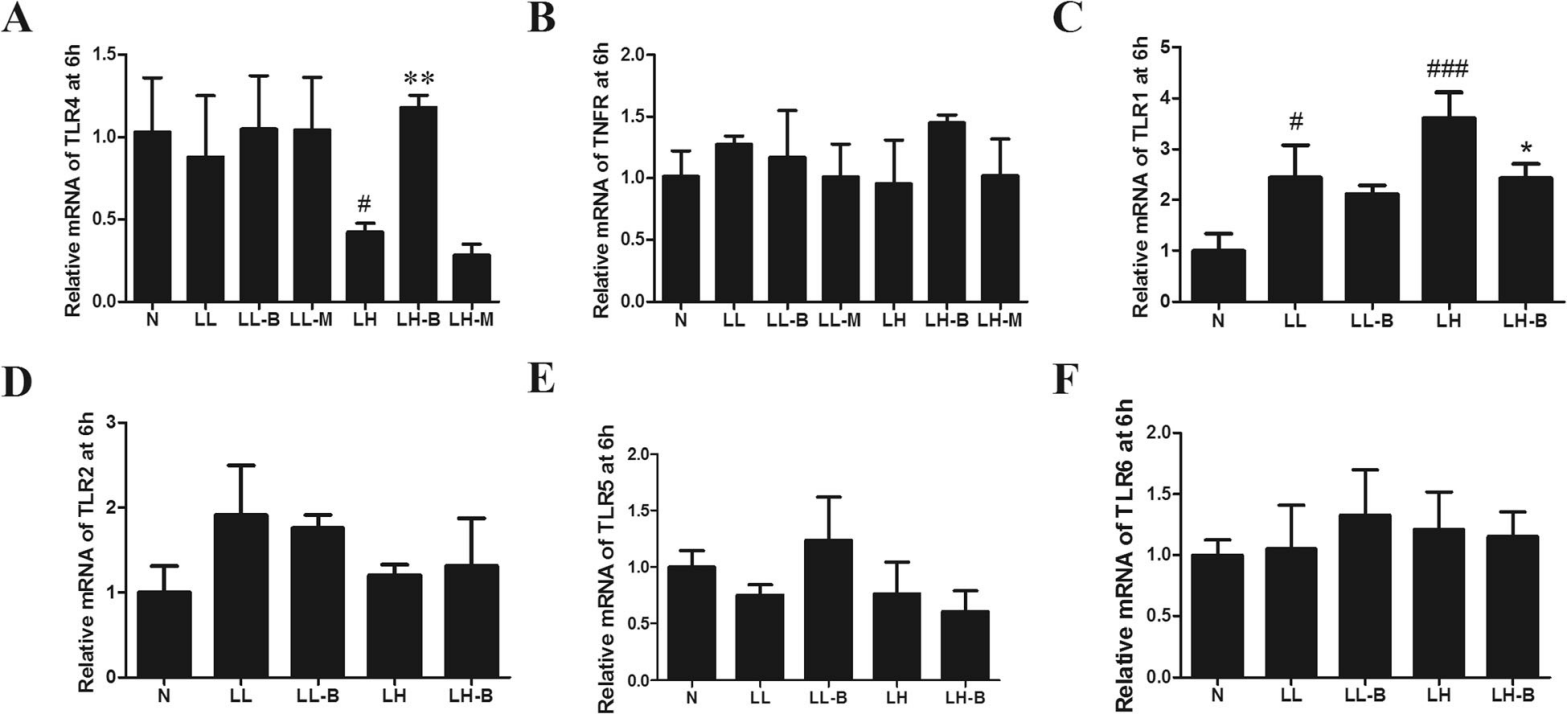
Effects of BBR on mRNA levels of inflammation-related proteins induced by LPS in Raw264.7 cells. (**a**) TLR4. (**b**) TNFR. (**c**) TLR1. (**d**) TLR2. (**e**) TLR5. (**f**) TLR6. N, normal control group; LL, low LPS group; LL-B, low LPS + BBR group; LL-M, low LPS + Metformin group; LH, high LPS group; LH-B, high LPS + BBR group; LH-M,high LPS + Metformin group; compared with N group, ^#^*P* < 0.05, ^###^*P* < 0.001; compared with LL or LH group, *P < 0.05, ***P < 0.001

**Fig. 9:**
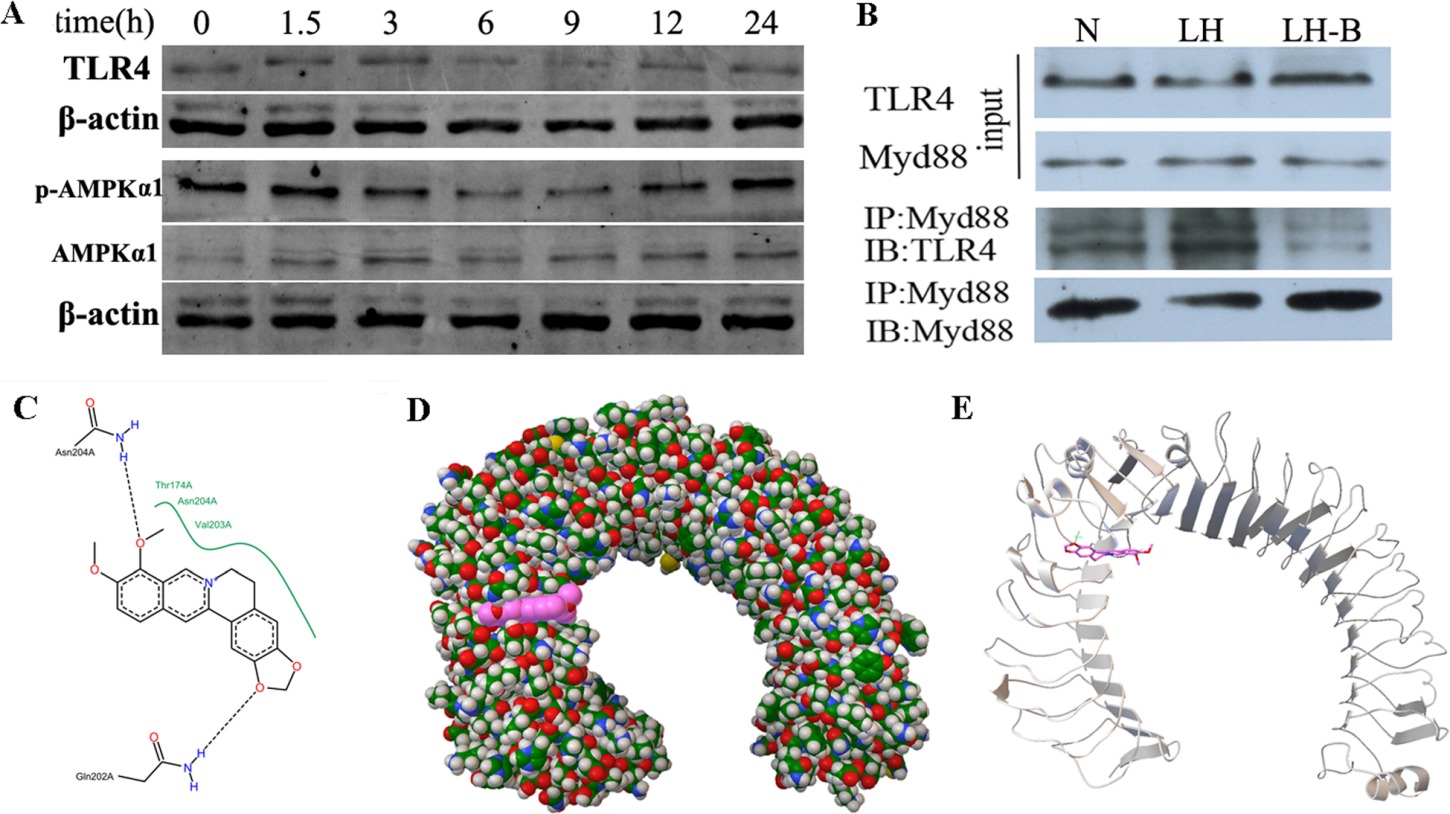
Effects of BBR on TLR4 and p-AMPK protein levels (**a**) and the interaction between TLR4 and MyD88 (**b**) induced by LPS in Raw264.7 cells. N, normal control group; LH, high LPS group; LH-B, high LPS + BBR group. **c**-**e** Molecular docking diagrams of the interaction between BBR and TLR4. **c** 2D docking diagram of the interaction between BBR and TLR4. The black dotted line and green curve indicate a hydrogen bond and hydrophobicity, respectively. BBR can form hydrogen bonds with Thr174, Asn204 and Val203, and can also form hydrophobic interactions with the Asn204 and Gln202 residues in the A chain of TLR4. **d** The surface structure of the ligand formed around the active centre of the A chain of TLR4. Red, BBR. **e** The conformation of the interaction between the ligand and the A chain of TLR4 after optimization

BBR had significant anti-inflammatory effects upon intervention with LPS for 3 h, and RT-PCR and Western blotting showed that BBR did not significantly improve the mRNA and protein levels of TLR4 at this time point (Figs. 8 and 9a). However, there were no significant differences in the secretions of TNF-α after pretreatment with AICAR or compound C or LPS stimulation for 3 h (results not shown). Meanwhile, agonists and inhibitors of AMPKα did not significantly improve the inflammatory response induced by LPS for 3 h. The expression levels of TLR4, p-AMPK and TNFR might not reflect the mechanism by which BBR inhibited M1 macrophage polarization after LPS intervention for 3 h.

### Effects of BBR on the interaction between TLR4 and MyD88

The interaction between TLR4 and MyD88 was detected by CoIP. The results showed that the interaction between TLR4 and MyD88 increased after LPS intervention for 3 h, and BBR inhibited the binding of TLR4 to MyD88 (Fig. 9b, Additional file 1: Figure S1). Based on structural biology, BBR interacted with the A chain, B chain and C chain of TLR4, and its affinity for the A chain binding site was the highest (Fig. 9c-e), the I conformation number was 100, and the docking free energy was − 6.69 kcal/mol.

## Discussion

M1 macrophages secreted inflammatory cytokines, such as TNF-α, IL-6, IL-1β, MCP-1 and MIF-1, which affected insulin sensitivity [24], and inhibiting inflammation can improve insulin function. Similar to previous studies, this study found that BBR inhibited M1 polarization. Notably, the inflammatory mediators were significantly increased after LPS treatment for 3 h; meanwhile, the anti-inflammatory effects of BBR were obvious. The production of these inflammatory factors was mainly promoted by the nuclear translocation of NFκB p65 [25], and the activation of NFκB in macrophages was regulated by many signalling pathways, such as pathways involving TLRs, AMPK α1 and TNFR [26–28]. Then we explored the mechanism by which BBR inhibited M1 polarization. However, the transcriptional changes in TNFR, TLR1, TLR2, TLR5 and TLR6 seemed not the answers. Consistent with the finding that agonists and inhibitors of AMPKα did not significantly improve the inflammatory response, the expression of p-AMPK did not change significantly after LPS intervention for 3 h.

TLR4 plays the most important role in macrophage inflammation mediated by LPS [29]. The specific knockout of TLR4 in myeloid cells inhibited insulin resistance in high-fat-diet-fed mice [30]. TLR4 can anchor MyD88 and promote the nuclear translocation of NFκB to induce the expression of inflammatory factors [31]. In fact, some studies have found that the anti-inflammatory effect of BBR is related to TLR4 expression [32, 33]. In this study, macrophage inflammation and anti-inflammatory effects of BBR appeared after LPS intervention for 3 h and TLR4 protein levels did not increase at the same time. Therefore, the mechanism underlying the action of BBR was further analysed based on structural biology. The results of CoIP and molecular docking showed that the affinity of BBR for TLR4 was high, and the interaction between TLR4 and MyD88 was inhibited by BBR. Thus, the inhibition of inflammation by BBR in the early phase may be related to its interaction with TLR4, which may interfere with the binding of MyD88 to TLR4.

One intriguing finding of this study was that BBR can interact with TLR4 to interfere with inflammatory signalling pathway. In fact, the TLR4 signalling pathway could also be regulated by many mechanisms. Inflammatory factors could induce the upregulation of TLR4 expression, while sTLR4, RP105, MyD88s, A20 and miRNA-21 were negative regulatory elements [34]. These negative regulatory factors could affect TLR4 through degradation, deubiquitination and competitive binding [35], and this may explain the decreasing trend of TLR4 after LPS treatment for 9 h. In addition, TLR4 is a transmembrane protein that consists of three parts: the extracellular, intracellular and transmembrane regions [27]. The extracellular segments are involved in the recognition of LPS and the intracellular segments are involved in signal transduction. BBR seemed to interact with TLR4 at several sites, and the precise function site needs to be further confirmed.

In conclusion, this study showed that BBR could inhibit the pro-inflammatory M1 polarization of macrophages in the early phase, and that the effects were related to interference in the process of TLR4 and MyD88 binding and the nuclear translocation of NFκB p65. Interference in the TLR4/MyD88/NFκB signalling pathway inhibits metabolic inflammation based on structural biology, which may be a new mechanism by which BBR prevents and treats T2DM.

## Conclusions

Inflammatory changes were induced in macrophages after LPS stimulation for 3 h, and BBR pretreatment inhibited inflammatory polarization. BBR could interact with TLR4 and disturb the signal transduction of the TLR4/MyD88/NFκB signalling pathway, and this might be the mechanism by which BBR attenuates inflammation in the early phase.

## Supplementary information

**Additional file 1:** **Figure S1.** Protein levels of TLR4 (A) and p-AMPK (B) and effects of BBR on combination of TLR4 and MyD88 in CoIP assay (C) induced by LPS in Raw264.7 cells. Compared with N group, ^#^*P* < 0.05, ^###^*P* < 0.001; compared with LH group, *P < 0.05.

## Data Availability

The datasets used and/or analysed during the current study are available from the corresponding author on reasonable request.

## Abbreviations

AMPK: Adenosine 5′-monophosphate (AMP)-activated protein kinase
BBR: Berberine
FBS: Fetal bovine serum
IL-1: Interleukin-1
IL-6: Interleukin-6
INOS2: Inducible nitric oxide synthase 2
LPS: Lipopolysaccharide
MIP1: Macrophage inflammatory protein 1
MyD88: Myeloid differentiation factor 88
NFκB: Nuclear transcription factor-κB
PBS: Phosphate buffered saline
T2DM: Type 2 diabete mellitus
TLR: Toll-like receptor
TNFR: Tumor necrosis factor receptor
TNF-α: Tumor necrosis factor α
