# Supplementary figures and images for: Inhibitory effects of berberine on proinflammatory M1 macrophage polarization through interfering with the interaction between TLR4 and MyD88
Source: BMC Complement Altern Med. 2019 Nov 19;19:314. doi: 10.1186/s12906-019-2710-6 (PMC6862859; doi:10.1186/s12906-019-2710-6)

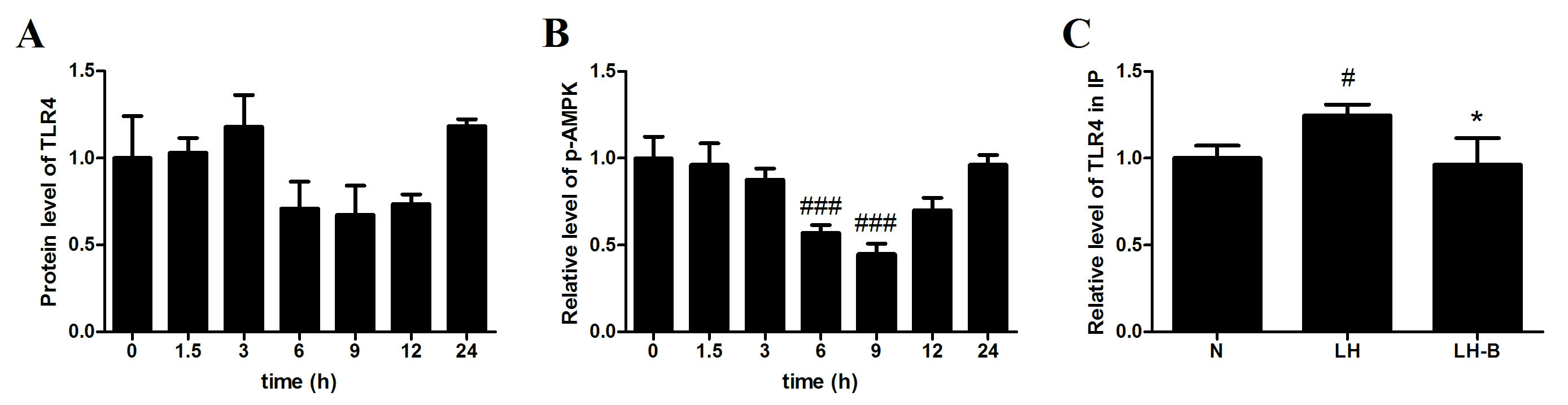

Supplement: Supplementary file 1 — Additional file 1: Figure S1. Protein levels of TLR4 (A) and p-AMPK (B) and effects of BBR on combination of TLR4 and MyD88 in CoIP assay (C) induced by LPS in Raw264.7 cells. Compared with N group, #P < 0.05, ###P < 0.001; compared with LH group, *P < 0.05. [file 12906_2019_2710_MOESM1_ESM.tif]
